# Indomethacin versus Ketorolac for the prevention of heterotopic ossification in hip arthroscopy patients

**DOI:** 10.1016/j.jor.2025.07.032

**Published:** 2025-07-29

**Authors:** Christopher S. Frey, Thomas M. Spears, Daniel Puczko, Alicia M. Hymel, Candler G. Mathews, Patrick M. Luchini, Katherine D. Van Schaik, Jessica R. Leschied, Jaron Sullivan

**Affiliations:** a Department of Orthopaedic Surgery, Vanderbilt University Medical Center, 1215 21st Ave S Suite 4200, Nashville, TN, 37232, USA; b Vanderbilt University School of Medicine, 340 Rudolph A. Light Hall, Nashville, TN, 37232, USA; c Center for Musculoskeletal Research, Vanderbilt University Medical Center, 1215 21st Ave S Suite 4200, Nashville, TN, 37232, USA; d Department of Radiology and Radiological Sciences, Vanderbilt University Medical Center, 1211 Medical Center Dr, VUH 1145, Nashville, TN, 37232, USA

**Keywords:** Femoroacetabular impingement, Hip arthroscopy, NSAID, Heterotopic ossification

## Abstract

**Background:**

Nonsteroidal anti-inflammatory drugs are commonly utilized to reduce the risk of developing heterotopic ossification (HO) after hip arthroscopy. However, it is not known which regimen is optimal.

**Purpose:**

The purpose of this study is to determine the rate of HO formation after hip arthroscopy in response to different NSAID protocols.

**Methods:**

Consecutive cases of a single fellowship-trained surgeon at a tertiary referral center were retrospectively reviewed. Patients received a regimen of two medications, starting with four days of either Ketorolac or Indomethacin and ending with either Celecoxib, Meloxicam, Diclofenac, or Naproxen. Two reviewers assessed HO on postoperative radiographs.

**Results:**

323 cases were included for retrospective review. 48 (15 %) were found to develop HO after hip arthroscopy. Patients who also underwent labral repair (p = 0.046) and those with larger corrections in alpha angle (p = 0.048) were found to have higher rates of HO. Multivariate regression found that receiving Meloxicam as a second medication was found to have a significantly higher risk of HO than Celecoxib (OR 4.72, p = 0.035). Male gender (OR 2.36, p = 0.013), was also found to be associated with a higher likelihood of HO formation according to the model.

**Conclusion:**

While taking Meloxicam as the second NSAID was associated with a significantly higher rate of HO than Celecoxib, no one regimen was found to be superior. Additionally, male gender was found to be a significant predictor of HO development.

## 1 Introduction

Hip arthroscopy is a relatively recent development within orthopedic surgery that has grown substantially as indications and techniques have evolved.^1^^,^^2^ In fact, one study found an annualized 30 % growth rate in surgeries from 2008 to 2012, and a more recent paper depicted an 85 % increase from 2011 to 2018.^2^^,^^3^ Although there has been demonstrated safety and efficacy of arthroscopic hip procedures, there does exist a low risk of complications.^4^ One complication, postoperative heterotopic ossification (HO) is often asymptomatic but may cause pain and stiffness, sometimes requiring excision.^5^ There is variable incidence reported in the literature ranging from 0 % and to as high as 44 %, although there is notable heterogeneity in screening and prophylaxis protocols.^6^, ^7^, ^8^

Postoperative heterotopic ossification is thought to be attributable to an inflammatory response to tissue injury.^9^ Proposed mechanisms involve the presence of osteogenic cells, vascular supply, and a local inflammatory cascade.^10^ In order to prevent this, prophylactic modalities should inhibit these pathways.^10^ The two main treatments used for prophylaxis are radiation and non-steroidal anti-inflammatory drugs (NSAIDs). NSAIDs are thought to inhibit progenitor cells from differentiating into osteocytes.^10^ Evidence has mounted such that prophylaxis with NSAIDs is now considered standard of care.^11^ One study found that patients who received Naproxen developed HO at a rate of 5.6 % compared to 25 % in the group without prophylaxis.^12^ Of those who did develop HO, 26 % of patients were symptomatic and required resection.

Although it is now well understood that NSAIDs are effective, it has not been determined which regimen is the most efficacious.^11^ Ascertaining the optimal prophylactic regimen would be valuable in optimizing outcomes of hip arthroscopy for femoroacetabular impingement (FAI). With a lower risk of gastrointestinal side effects, selective COX-2 inhibitors may present an attractive option. Although this class has been shown to have similar efficacy in total hip arthroplasty, the data is not quite as robust for hip arthroscopy.^13^^,^^14^ The goal of this study was to analyze the impact of different NSAID regimens on the incidence of HO after hip arthroscopy for HO. We hypothesized that patients receiving Indomethacin and Naproxen would have lower rates of HO.

## 2 Methods

After standard institutional review board approval, a retrospective review was performed on all hip arthroscopies performed by the senior author at a single institution from November 2014 to April 2024. The CPT codes 29914, 29915, and 29916 were used to identify patients. Inclusion criteria consisted of preoperative and post-operative pelvis radiographs at least 3 months out from surgery, one of the aforementioned hip arthroscopy procedures, and having received postoperative HO prophylaxis with NSAIDs. Exclusion criteria included prior heterotopic ossification, inadequate imaging, and concomitant major surgery, such as an osteotomy.

### 2.1 Patient evaluation

Data was extracted from patient encounters and images in the electronic health care records. Standard radiographs consisted of a preoperative and postoperative series including low AP pelvis, false profile, frog leg lateral, and a modified Dunn view. Patients were scheduled for routine follow-up at 2 weeks, 6 weeks, 3 months, 6 months, and 1 year. Imaging was obtained routinely at the 3 month visit. If patients had adequate hip radiographs from a later date, this was used for analysis. Radiographs were analyzed by a musculoskeletal radiology attending, orthopaedic sports fellow, and/or a medical student.

The alpha angle was measured using the modified Dunn view.^15^^,^^16^ Heterotopic ossification was graded using the Brooker classification, which has previously been applied to hip arthroscopy.^12^^,^^17^ In cases of disagreement, as defined by any difference in Brooker grade or a difference in alpha angle measurement of 7° or more (2 SD), a tiebreaker measurement was performed by one of the orthopaedic sports fellow reviewers who had not recorded one of the 2 measurements. For alpha angle measurements, the average of the two closest measurements was used, and the third measurement was discarded. In cases where the alpha angle differed by 6° or less, the average of the initial two measurements was used. For the Brooker classification, in all cases for which a third reviewer was required, the tiebreaker classification agreed with one of the two initial reviewers, and the discrepant classification was discarded.

Alpha angle measurement Intraclass Correlation Coefficients (ICC) were calculated using two-way random effects models based on rater agreement with the intent of using the average of the ratings as the unit of analysis.

### 2.2 Surgical technique and postoperative rehabilitation protocol

All procedures were performed by one fellowship trained surgeon at a tertiary referral center (J.P.S.). Briefly, patients underwent general anesthesia and were placed supine on a post-less traction table. Access was typically obtained through three portals. This consisted of the anterolateral, anterior, and distal anterolateral accessory (DALA) portals. After the anterolateral and anterior portals were established, an interportal capsulotomy was created. An acetabuloplasty was performed in the presence of pincer-type pathology, and either debridement, repair, or allograft reconstruction was used for labral pathology per the surgeon's discretion. Next, traction was removed, and a T-capsulotomy was performed. Femoroplasty was performed for cam-type impingement. Both capsulotomy limbs were repaired at the end of the case. A local anesthetic cocktail consisting of ropivacaine, epinephrine, clonidine, and ketorolac was injected into the joint prior to closure.

Beginning the day after surgery, patients received four days of one of two initial medications: Indomethacin 75 mg QD or Ketorolac 10 mg QID. This was followed by 30 days of one of four medications: Naproxen 500 mg BID, Meloxicam 15 mg QD, Celecoxib 200 mg BID, or Diclofenac 75 mg BID. In total, 8 combinations of prophylactic medications were prescribed, which changed over the course of the senior author's practice. Patients initially received Indomethacin but it was not always available at the local pharmacy, so this was switched to Ketorolac.

After surgery, patients were permitted to bear weight as tolerated without a brace. They were instructed to ambulate with crutches for the first 2 weeks to reduce the risk of falling. Rehabilitation was initiated 1–3 days postoperatively, focusing on range of motion. Patients then progressed through a five-phase protocol that lasted for 16 weeks.

### 2.3 Statistical analysis

Descriptive statistics were used to summarize patient characteristics. Categorical variables were reported as frequencies and percentages, and continuous data were reported as means and standard deviations. Bivariate analyses of differences in characteristics between patients who did and did not develop HO were conducted, including Wilcoxon rank sum test for continuous variables and Fisher's exact test or Pearson's Chi-squared test for categorical variables. Multivariate logistic regression analyses were conducted to assess whether NSAID regimens were predictive of HO while controlling for sex, prior hip surgery, acetabuloplasty, and the pre-to post-operative degree change in alpha angle. Model covariates were selected a priori based on known associations with HO. Two separate models were performed. The first model used the first NSAID administered as a covariate, and the second model used the second NSAID administered as a covariate. Both first and second NSAID were unable to be included in the same model due to the number of HO cases in the sample. All analyses were run in R version 4.2.

## 3 Results

**Fig. 1:**
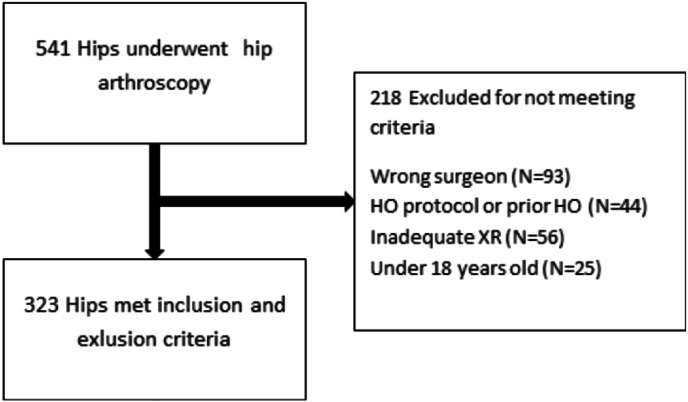
Patient flowchart.

In total, 507 patients and 542 operative hips were identified. After exclusion criteria, there were 323 (59 %) hips eligible for analysis (Fig. 1). The average age was 32.4 years (10.1) and average BMI 27.7 (4.9). 52.9 % of the cases were female (Table 1). A high degree of reliability was found between the measurements. The ICC for the initial two alpha angle raters was 0.96 (0.96–0.97 95 % CI), and the ICC for the final two ratings used to calculate the average was 0.99 (0.98–0.99 95 % CI). Cohen's Kappa for the initial two Brooker classification raters was 0.86, with perfect agreement between the final two ratings after the third rater was utilized.

**Table 1:** Patient characteristics by HO.

Characteristic	Overall, N = 323^a^	No HO, N = 275^a^	Any HO, N = 48^a^	p
Age	32.4 (10.1)	32.3 (10.3)	32.9 (9.3)	0.461
BMI	27.7 (4.9)	27.5 (5.0)	28.4 (4.6)	0.089
Gender (% Male)	152 (47.1 %)	121 (44.0 %)	31 (64.6 %)	**0.008**^b^
Prior hip surgery	27 (8.4 %)	23 (8.4 %)	4 (8.3 %)	>0.999 c
Alpha angle change (degrees)	17.5 (10.3)	17.0 (10.2)	20.4 (10.9)	**0.048**
Current smoker	38 (11.8 %)	29 (10.6 %)	9 (18.8 %)	0.106 b

a N (%) or Mean (SD). Missing data was present in the following variables: BMI (1), Current Smoker (1). Continuous variables were analyzed using Wilcoxon rank sum test. Categorical variables were analyzed using.

b Pearson's Chi-squared test or.

c Fisher's exact test.

Bivariate analyses revealed that men (p = 0.008) and patients with larger corrections in alpha angle were significantly more likely to develop HO (p = 0.048). While all patients received hip arthroscopy with femoral osteoplasty, 37 (11.5 %) also received acetabuloplasty and 261 (80.8 %) underwent labral repair (Table 2). Labral repair (p = 0.046) and larger changes in the alpha angle (17.0 vs 20.4, p = 0.048) were significantly associated with development of HO.

**Table 2:** Summary of procedures performed.

Procedure	No HO, N = 275^a^	Any HO, N = 48^a^	p
CPT 29915: Acetabular osteoplasty	35 (12.7 %)	2 (4.2 %)	0.136
CPT 29916: Labral repair	217 (78.9 %)	44 (91.7 %)	**0.046**
CPT 29861: Loose body removal	19 (6.9 %)	5 (10.4 %)	0.375
CPT 29862: Microfracture	14 (5.1 %)	1 (2.1 %)	0.708
CPT 29999: Arthroscopic psoas tenotomy OR Labral reconstruction	33 (12.0 %)	4 (8.3 %)	0.625
CPT 27299: Core decompression of femoral head	1 (0.4 %)	0 (0.0 %)	>0.999
CPT 29863: Debridement of the ligamentum teres	3 (1.1 %)	0 (0.0 %)	>0.999

a N (%). Categorical variables were analyzed using Fisher's exact test.

The postoperative radiographs reviewed for analysis were obtained at a median 174 days after surgery. A total of 48 (14.9 %) developed heterotopic ossification to some degree. Of these hips, 39 (81.3 %) were Brooker 1, 6 (12.5 %) were Brooker 2, and 3 (6.3 %) were Brooker 3. 4 complained of pain or limitations to ROM, 3 of impingement, and 4 returned to the OR for HO excision.

There were 8 combinations of NSAID prophylaxis utilized over the course of the study. Multivariate regression analyses were run to determine which NSAID regimens were predictors of HO. The first administered NSAID, Ketorolac or Indomethacin, was not significantly predictive of the presence of HO. However, receiving Mobic as the second medication resulted in a significantly higher rate of HO compared to Celecoxib (OR 4.72, p = 0.035). No other adjusted pairwise comparisons achieved significance (Table 3).

**Table 3:** Pairwise contrasts of adjusted odds of developing HO.

Contrast	Adjusted OR	SE	p a
Diclofenac/Celecoxib	4.44	3.65	0.070
Naproxen/Celecoxib	2.09	0.93	0.099
Meloxicam/Celecoxib	4.72	3.48	**0.035**
Naproxen/Diclofenac	0.47	0.35	0.307
Meloxicam/Diclofenac	1.06	1.00	0.948
Meloxicam/Naproxen	2.26	1.46	0.209

a All p values are unadjusted for multiple comparisons.

Analysis of other covariates found that male gender was significantly associated with the development of HO (First NSAID Model: OR 2.36, p = 0.013; Second NSAID Model: OR 2.15, p = 0.026), mirroring the unadjusted analysis. Neither prior hip surgery, nor presence of acetabuloplasty were found to have a significant association with finding the development of HO postoperatively.

## 4 Discussion

Although it is common to employ anti-inflammatories for HO prophylaxis after hip arthroscopy, there is no consensus regimen. Such knowledge may be valuable in helping guide the development of this rapidly evolving field. The primary goal of this study was to determine the impact of different NSAID protocols on the development of HO after hip arthroscopy for CAM impingement. Retrospective analysis found that 15 % of patients developed some degree of HO. Only 8.3 % (1.2 % overall) of these patients were symptomatic. This is in line with the range produced by prior studies.^18^ Additionally, male gender and receiving Meloxicam as the second medication were associated with a higher odds ratio of having HO detected on postoperative imaging.

When comparing Meloxicam to Celecoxib as a second medication, Meloxicam was found to have a higher risk of HO, however, there were no other differences detected between the prophylactic NSAID regimens. A recent meta-analysis of hip arthroplasty found that selective NSAIDs as a class did not outperform non-selective NSAIDs.^19^ However, celecoxib was associated with the lowest rate of heterotopic ossification out of the 10 medications analyzed. This may be attributable to an improved therapeutic range given lower risk of GI side effects.

Out of multiple covariates assessed, male gender was found to be significantly associated with heterotopic ossification. Prior hip surgery, the presence of acetabuloplasty, and the change in alpha angle were not found to significantly impact the likelihood of developing HO in our regression. In mouse models, male gender has been found to be associated with burn induced HO formation, however, data in humans is mixed.^20^ Bedi et al. found that the vast majority of patients who developed HO and patients who required excision of HO were male.^21^ Dow et al., however, did find that males had a significantly higher rate of HO after hip arthroscopy.^22^ In contrast, several studies have failed to establish any role of gender in the development of HO. ^6^^,^^12^ More research is required to fully assess the risk of gender.

In addition, bivariate analysis found that concomitant labral repair was significantly associated with the development of HO. There is limited data on this relationship. Mortenson et al. found that neither labral repair nor the number of repair anchors were significantly associated with HO formation in patients who received Naproxen for prophylaxis.^23^ Bedi et al. compared patients who received indomethacin followed by Naproxen with those who received Naproxen alone. Although the indomethacin group had a repair rate of 62.8 % as opposed to 10.5 % in the Naproxen alone group, the rate of HO in the dual medication group was found to be significantly lower.^21^ It is conceivable that the additional time of surgery, bone or periosteum particles, and soft tissue manipulation may contribute to the development of HO. Of note, labral repair was not included in the regression model because the variables were determined a priori. When labral repair was included, it did not significantly change any of the other outcomes.

Interestingly, a greater change in alpha angle from preoperative to postoperative measurements was detected in the bivariate analysis, however it did not significantly predict HO development in the multivariate regression. Similarly, acetabuloplasty was not determined to be a significant factor despite these procedures also increasing time and bony debris. Beckman et al. found that both having a mixed resection and a larger change in alpha angle were independently associated with forming HO.^12^ Perhaps there is not a simple linear relationship between the volume of particulates generated and development of HO.

There are limitations of the study worth considering. Only 323 of 542 (60 %) hips initially screened met inclusion and exclusion criteria. It is possible that there may be selection bias in those who were ultimately included. 8 different HO prophylaxis regimens were implemented as the senior author's surgical practice evolved. Thus, the medication regimens were not randomized or evenly distributed. This study was also not able to assess if the patients truly completed the medication regimen. Although Meloxicam was associated with a higher rate of HO, the total number of patients on meloxicam was low. Lastly, as this study spanned nearly 10 years, surgical techniques may have changed as well.

## 5 Conclusion

This study found that 15 % of patients who received NSAID prophylaxis after undergoing hip arthroscopy with cam lesions developed HO. However, only 1.2 % of patients were symptomatic. No one regimen was found to be superior. Male gender, change in alpha angle, and labral repair were found to significantly increase the risk of HO. However, only male gender was found to predict HO in the multivariate analysis.

## Author contributions

Christopher S Frey MD- Data curation, supervision, writing-original, writing-review and editing.

Thomas M Spears DO- Conceptualization, methodology.

Daniel Puczko BA- Data curation, writing-review and editing.

Alicia M Hymel MS- Data curation, formal analysis.

Candler G Mathews MD- Data curation, writing-review and editing.

Patrick M Luchini MD- Writing-review and editing.

Katherine D Van Schaik MD, PhD- Data curation.

Jessica R Leschied MD- Data curation.

Jaron Sullivan MD- Conceptualization, writing-review and editing.

## Consent

This study was reviewed and approved by the institutional review board on 3/13/2024. It received IRB # **240434.** It qualified as secondary research for which consent is not required. (45 CFR 46.104) (d) (4).

## Ethical statement

This study was reviewed and approved by the institutional review board on 3/13/2024. It received IRB # **240434**.

## Funding/sponsorship

This study did not receive any grants.

## Funding statement

This research did not receive any specific grant from funding agencies in the public, commercial, or not-for-profit sectors.

## Declaration of competing interest

The authors declare that they have no known competing financial interests or personal relationships that could have appeared to influence the work reported in this paper.
